# Eye-Tracking Evaluation of Exit Advance Guide Signs in Highway Tunnels in Familiar and Unfamiliar Drivers

**DOI:** 10.3390/ijerph18136820

**Published:** 2021-06-25

**Authors:** Ting Shang, Hao Lu, Peng Wu, Yi Wei

**Affiliations:** 1 School of Traffic & Transportation, Chongqing Jiaotong University, Chongqing 400074, China; shangting@cqjtu.edu.cn (T.S.); 622190950074@mails.cqjtu.edu.cn (P.W.); 2 School of Economics & Management, Chongqing Jiaotong University, Chongqing 400074, China; 622190122033@mails.cqjtu.edu.cn

**Keywords:** exit advance guide sign, eye-tracking evaluation, familiar and unfamiliar drivers, highway tunnel safety, Markov chain

## Abstract

As a component of the traffic control plan, traffic signs on highways offer drivers necessary information. Unfortunately, many signs are unfamiliar to or misunderstood by drivers, especially when lacking a setting method; this includes exit advance guide signs in tunnels. These are generally set in roadbed sections, but space limitations in tunnels dictate that they must be set differently. To evaluate the effect of the setting method, an experiment was designed and conducted, during which the eye movements of 44 drivers with different familiarity levels were tracked. Twenty-two of the drivers had not previously participated in any experiment involving exit advance guide signs in highway tunnels, while 22 of them had. Time period data were analyzed, including data from before the sign appeared, when it appeared, and when it disappeared. Based on area division and Markov theory, attributes related to gaze transition were obtained, including one- and two-step gaze transition probabilities and area gaze probabilities. The results showed that gaze transition was confirmed to be significantly different between the three periods and between the drivers. Features extracted from eye movement characteristics, gaze transition paths, and gaze areas demonstrated that visual attention is more dispersed in familiar drivers during the lane-change intention period. Therefore, signs should be placed on the left wall of the highway tunnel.

## 1. Introduction

Traffic signs are essential components of the traffic control plan. They offer drivers necessary information on upcoming situations [1,2,3], thus playing a key role in highway safety. Highway construction is extending to western mountains areas in China, so the number of tunnels is increasing. Tunnels can even constitute as much as 90% of a highway, with only 10% being roadbed. Tunnels comprise a significant section of the highway bottleneck, especially in mountains, and the safety performance in these areas is critical. However, visual problems in tunnels often arise because of the poor lighting and the limitations of closed space. Research has indicated that the rate of traffic accidents is lower in tunnels than on highways, but that the accident fatality rate is much higher [4,5]. For example, a traffic accident occurred in the Shanxi Yanhou tunnel in China on 1 March 2014, causing the deaths of 40 people and injuring 12 [6]. Another traffic accident occurred at the Hachihonmatsu tunnel in Japan on 17 March 2016, causing the death of two people and the injuring 70 people [7].

The black- or white-hole effect results in the poor discrimination ability of the driver upon exiting. Frequent lane changes and traffic flow interweaving in connecting segments of tunnels and interchanges can easily cause traffic accidents. Therefore, the effect of exit advance guide signs in highway tunnels is closely related to traffic safety.

Exit advance guide signs in tunnels must be easily visible, intuitive, and understandable [8]. Driver comprehension of signs generally differs. For example, of the 85 symbols included in the USA Manual on Uniform Traffic Control Devices (MUTCD), U.S. drivers showed comprehension levels ranging from 99% to below 40% for around 10 signs [9]. This may be due to the process of visual adaptation, where the visual attention system will cease to register the presence of a traffic sign that is often repeated [10]. Low sign comprehension levels were found among drivers who had not previously participated in a study compared with drivers who had previously participated in a study; hence, the former need more time to recognize and comprehend the meaning of a sign than the latter [9,11,12]. The United Nations World Tourism Organization recorded 1.2 billion international tourist arrivals worldwide in 2015 [13], a large number of whom rented vehicles during their stay. Driving in unfamiliar environments can increase the rate of road traffic accidents. Aty and Radwin [14] found that such drivers have higher accident rates. Traffic signs are designed to provide critical cues for drivers, regardless of where they are from. To ignore or misunderstand this information can cause errors in judgment and increase the risk of accidents [15]. Much research has been carried out on driver behavior and safety, but little has been conducted from the perspective of an unfamiliar driver’s perspective.

To reduce traffic accidents, we researched the effect of exit advance guide signs in highway tunnels on the behavior of drivers of levels of different familiarity. Few studies have discussed the methods for setting up such signs for tunnel exits on highways. We proposed [16] the use of separate guide signs in tunnels, which has great potential to improve the safety of highway tunnel-interchange sections. Exit advance guide signs include location and distance signs, which are set separately. Location signs use Chinese characters with a height of 40 cm and a width of 20 cm, which are set on the tops of tunnels. Distance signs use Chinese characters with a character height and width of 1.5 m, a character spacing of 1.5 m, and total sign length of 22.5 m, and are set on the sides of tunnels. The objective of this study is to analyze the impact of exit advance guide sign settings in tunnels on the driver’s eye tracking. Using experimental simulation data from 44 participants, the eye-tracking profiles of drivers with different levels of familiarity were analyzed. We used a Markov chain, including one- and two-step gaze transition probabilities and area gaze probabilities, to determine the gaze transfer regularity and stationary distribution and evaluate the effect of exit advance guide sign placement. The disparities between the groups reflect the risks associated with different types of drivers. This research work provides the basis for exit advance guide sign design in highway tunnels.

## 2. Literature Review

### 2.1. Effect of Familiarity on Driver Eye-Tracking

Driving is a complex task that includes the simultaneous execution of several motor and cognitive processes while moving through an environment filled with different types of information [17]. Road design guidelines should consider users who are driving on a roadway for the first time and are unfamiliar with its features [18]. The Highway Capacity Manual [19] suggests taking into account the vehicular composition of traffic flow with regard to route familiarity for the calculation of the equivalent flow for multilane highway/freeways. While unfamiliar drivers drive at a lower speed, this may be related to an increased accident risk [20]. The widely studied issue of driver distraction can be strongly linked to route familiarity. Shrira [21] argued that different driving environments have unique risks, and drivers in unfamiliar settings may not adapt to them. A driver’s unconscious eye-tracking can express the visual information processing procedure [22,23,24]. Stephanie Hurtado [25] explored drivers’ behavior when encountering road signs in three countries and found that participants spent increased time looking at unfamiliar road signs. The examination of the cross-cultural understanding of road signs revealed low comprehension scores for nonlocal signs and the misinterpretation of road sign rules [9]. Drivers who identify as having a poor sense of direction require additional support on unfamiliar routes [26,27]. While deliberation is effortful and occurs in unfamiliar situations, such as when learning to drive, habits are developed over time with repeated exposure to similar situations [28].

The literature shows that traffic signs are not always clear to all drivers, whose comprehension levels vary [8]. A follow-up study tested the relationship between sign comprehension and the extent to which signs comply with the ergonomic design principles of compatibility, familiarity, and standardization [12]. Babić [29] investigated the effect of the compliance of international traffic signs to principles of physical and conceptual compatibility and participants’ ability to learn unfamiliar signs. The results showed that ergonomically designed signs are easily understood and easily learned by those unfamiliar with them. Various studies have analyzed the effect of route familiarity on driver behavior and shown that, as drivers become more familiar with a roadway section, they choose higher speeds, shorten their glances at signs, and react more slowly to stimuli in their peripheral vision [30,31]. Martens [32] conducted a driving simulation experiment whose participants drove through a course 24 times over five consecutive days, with a change in a key traffic control sign occurring at one intersection during the final test run. Familiarization was found to occur rapidly during the first few repetitions.

The above-cited research used eye tracking to determine gaze behavior; further research is needed on traffic sign comprehension, especially in tunnels.

### 2.2. Effect Setting Method of Exit Advance Guide Signs on Highways

An exit advance guide sign is an important piece of road infrastructure that indicates an exit is imminent, warning drivers to decelerate and prepare to change lanes. Zwahlen [33] investigated the effectiveness of ground-mounted diagrammatic advance guide signs in the context of urban multilane arterials leading to freeways. Based on auto navigation data, Song [34] developed the concept of an acceptable guiding level and a model of minimum guiding distance that can be used to calculate the range of the layout of advance entrance direction signs. Huang [35] simulated five design schemes of exit advance guide signs and found the optimal one under different outlet spacings. Liu [36] proposed an optimization model for current guide sign systems in the situation of adding a road. The model was applied in Guangzhou, which improved the accessibility from 77.9 to 88.9%. A speed limit sign location model was established by exploring a driver’s visual recognition process; recommended values for locating speed limit signs under different driving conditions were given [37]. Most of these studies did not consider the tunnel environment.

A tunnel is not only the throat of a highway but a place with a high incidence of traffic accidents. Tunnel accidents can easily cause serious casualties and rescue is difficult [38]. Tunnels in mountainous areas are characterized by shorter roadbed sections, which necessitates the placement of some exit advance guide signs in tunnels. However, the current criteria [39,40,41,42] and current guidelines provide no clear setting method, and the matter of how to set up exit advance guide signs effectively in tunnels is becoming a major issue. Some scholars have studied the setting of exit advance guide signs in tunnels. Wang [43] studied highway tunnel traffic signs for feature extraction and selection based on environmental factors and used a decision tree classification algorithm to simplify this complex problem. The experimental results showed good recognition results. Upchurch [44] analyzed the effects of different sign setting schemes in the 2.4 km (1.5 mi) Central Artery/Tunnel. Based on ergonomics and traffic theory and using the rate of change in the pupil area, Yan Bin [45] proposed a model for setting traffic signs in adjacent tunnel groups for safety. Song [46] obtained eye movement and motion data for a 380 m-long short tunnel where three layouts of signs and markings were installed.

However, highway tunnels and urban tunnels are quite different in terms of traffic volume, traffic composition, and illumination. In particular, tunnel groups on mountainous highways are more affected by the black- or white-hole effect and have a greater impact on a driver’s vision and driving characteristics. Therefore, it is necessary to discuss the effects of exit advance guide signs in highway tunnels.

### 2.3. Application of Markov Chain in Traffic Engineering

The Markov chain is a stochastic system undergoing transitions between states. Liechty [47] used hidden Markov models (HMMs) to differentiate local and global covert visual attention states from eye movement data. Instead of applying the Markov model to transition matrices, as we do in this article, HMMs were used to distinguish fixations. Krejtz [48] presented a two-step method to quantify eye movement transitions between areas of interest (AOIs).

A Markov chain model is often used in the data analysis of traffic gazes. To identify lane change intention, Peng [49] conducted driving experiments with 16 drivers and analyzed their eye movement characteristics using the Markov chain model. The results showed significant differences in the saccadic speed, eye–head coordination mode, and other parameters between the data for 5 s before lane change and 5 s during the lane-keeping period. Yan [50] proposed an HMM to connect driving risk, physiological information, and vehicle dynamic data. Using data from physiological measurement sensors, the model could identify dangerous and normal driving states and predict the probability of transition from dangerous to normal. Zhou [51] proposed a visual search stability evaluation method based on the characteristics of eye movement using a Markov chain by collecting visual and physiological data from 16 participants as they passed through 13 urban tunnels. Guo [52] studied the gaze transfer characteristics of drivers with different levels of experience in an urban tunnel environment. Wen [53] showed that drivers of cars and large trucks looked straight ahead more than 45% of the time. When turning left, the gaze strategy in different highway tunnel sections varied greatly when turning right, it was more stable.

In summary, while only a few scholars have researched exit advance guide signs, even fewer scholars have researched the setting effect of exit advance guide signs. If an exit advance guide sign is set on top of a tunnel, the limitations of tunnel clearance make it necessary to reduce the font size. This satisfies information integrity requirements but reduces recognition. Generally, in China the left lane is for light motor vehicles such as cars, and the right lane is for heavy motor vehicles such as trucks; hence, if a traffic sign is installed at the end of a parking lot, heavy motor vehicles would obstruct it from being viewed by light motor vehicles. A driver who is in this tunnel for the first time may miss or misunderstand the content of an unfamiliar sign. Therefore, it is necessary to study the effect of setting the front sign at the exit of the tunnel on familiar and unfamiliar drivers. Ergonomics is a primary means of studying the effects of traffic signs on driving behavior based on physical characteristics such as a driver’s vision [54]. Research on traffic signs using drivers’ visual characteristics is unanimously recognized as being of great importance. Scholars believe that the setting of advance guide signs should consider the visual characteristics of drivers, and eye trackers have been widely used to test drivers’ visual recognition in a traffic environment.

This paper proposes the use separate guide signs in tunnel exits that are placed using an eye tracker and simulation test platform. By changing the placement positions of signs, their setting effects on familiar and unfamiliar drivers can be obtained.

## 3. Methods

Driver behavior was tested using two schemes by means of a driving simulator, which can create nearly any driving scenario and collect vehicle speed and position data at a resolution not easily obtained in a real driving environment. Accompanying the simulator was a device used to track and record participants’ gazes, allowing the in-depth analysis and investigation of driver eye movement and behavior. The driving simulator is valid when compared to real world conditions.

### 3.1. Participants

A total of 44 participants, including 19 females and 25 males, participated in the experiment. The participants were between 22 and 58 years old, with an average age of 37 years and a standard deviation of 8.1 years. All the participants held a valid driver’s license in China. Half of them had previously driven in an experiment involving exit advance guide signs in highway tunnels. All provided informed consent and had normal or corrected-to-normal vision.

### 3.2. Experimental Apparatus and Driving Scenario

**Figure 1:**
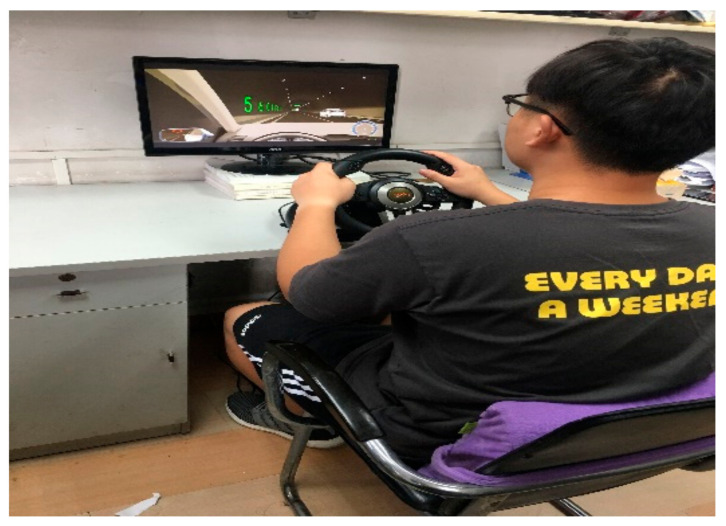
Driving simulator.

During the experiment, gaze position data were collected by the Tobii X2-60 eye tracker, which was produced by Tobii Electronic Technology (Suzhou, China) Co. Ltd. The sampling rate was 60 Hz and the accuracy was 0.34°. Velocity data were collected by a driving simulator included in the UC-win/road simulation software, which was produced by the Japan FORUM 8 Company (Tokyo, Japan), and a driving operating system produced by PXN-V3 Pro, ShenZhen PXN Electronics Technology Co. Ltd. (Shenzhen, China), as shown in Figure 1. The software included “Terrain Input”, “Road Definition”, “Road Generation and Traffic Flow Generation”, and “Edit, Output, Virtual Reality Simulation”. The sampling rate was 15 Hz. The driving operating system included a steering wheel, pedals, gears, and a handbrake controller. The noncontact design was easy to use, performed stably, and has been widely used in universities and research institutions.

### 3.3. Experiment Design

The proposed guide signs [16] included location and distance signs. The location sign was set in the middle of the top of the tunnel. According to the Specification for the Design of Highway Tunnels [39] and Road Traffic Signs and Markings [40], considering that traffic signs in a tunnel are affected by their head room, the height chosen was 40 cm, which is related to speed. Distance signs were placed 62.6 m from location signs, with a character height and width of 1.5 m and a total sign length of 22.5 m.

The experiment was set on a 4-lane dual highway with an 80 km/h design speed, which conforms to the design of most of China’s mountainous highways. Drivers were required to drive below 80 km/h and leave the highway after seeing the exit sign for the destination. The traffic volume was set to 1400 pcu/h, which is the maximum secondary service level. After one participant completed a test scene, another participant continued with the test. Participants stopped for 15 min between each testing scheme. This study did not consider the impact of different distance signs on the driver when driving in the right-lane scheme, because most cars drive in the left lane in China according to the Law of the People’s Republic of China on Road Traffic Safety. In addition, traffic rules do not allow lane changes in a tunnel. If the car is driving in the left lane, it must change lanes when out of the tunnel, but the car in the right lane is not required to do this. Therefore, we only simulated driving in the left lane. To increase the similarity with the real driving environment, we chose a traffic volume parameter which was set to 1400 pcu/h for testing.

### 3.4. Data Collection and Analysis

The effect of traffic sign setting was analyzed by the rate of change of the driver’s pupil area and the shift of the gaze point. From the perspective of the driver’s fixation transfer characteristics, the position of the fixation point at the current moment was only related to that at the previous moment. Therefore, we used a Markov chain to analyze all schemes [46,47,48,49,50,51,52].

## 4. Results

### 4.1. Gaze Transition Probability Analysis

#### 4.1.1. Area of Interest Division

**Figure 2:**
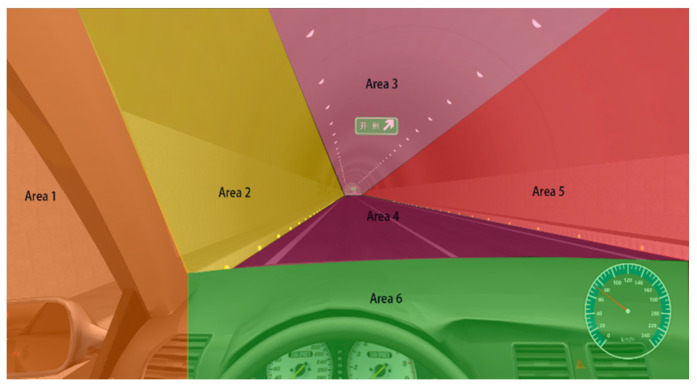
Areas of interest.

A participant’s field of vision (FOV) was divided into six areas, as shown in Figure 2 and listed in Table 1. Area 1 was the left side of the visual field, area 2 was the front-left of the visual field, area 3 was directly above the visual field, area 4 was the road surface, area 5 was the front-right of the visual field, and area 6 was the cab area, as shown in Table 2.

#### 4.1.2. Analysis of Gaze Distribution Characteristics

**Figure 3:**
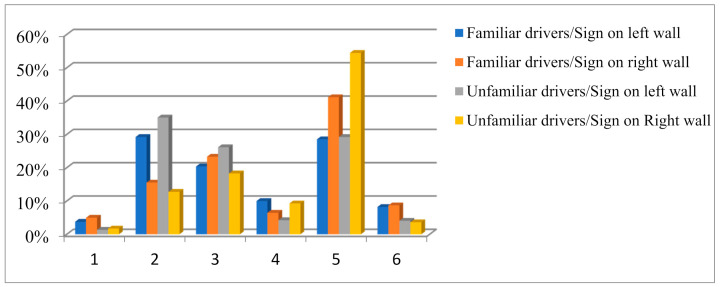
Gaze distributions of drivers with different familiarity levels.

By changing the locations of the distance signs, the proportions of points fixated on in each area can be obtained, as shown in Figure 3. By analyzing the significance, we found that the points focused on by familiar and unfamiliar drivers differed greatly. When a vehicle drove in the left lane and the distance sign was on the left wall, drivers paid the most attention to area 2 because the sign’s placement attracted their attention. When the distance sign was on the right wall, the familiar driver paid the most attention to area 5, while an unfamiliar driver paid more attention to area 2 and area 5 than a familiar driver would. As the right wall was set with a distance sign, the amount of attention the driver gave it was significantly increased, and the effect of the setting of a distance sign was found to be significant.

### 4.2. Gaze Transition Mode Based on Markov Chain

Before and after the distance sign appeared, there was a shift in the driver’s gaze point in each area of interest. According to classical probability, the shift probability was approximated by its frequency. Combined with the data on the drivers’ different gaze areas, the fixation data of the two schemes were statistically analyzed, and the one-step transfer probability matrix of the drivers’ gaze points was obtained in three stages—i.e., before the sign appeared, when it appeared, and after it disappeared—so as to analyze the transfer characteristics of drivers’ gaze points.

#### 4.2.1. One-Step Gaze Transition Probability Matrix

The one-step gaze transition probability was used to analyze changes in adjacent gaze points. The fixation data of 22 familiar drivers and 22 unfamiliar drivers were selected for statistical analysis, and the mean value was taken in combination with the drivers’ different fixation areas. The statistical estimation method was used to solve the one-step transition probability matrix of the drivers’ fixation points in three sections of the tunnel; the results are shown in Table 1. The rows of each matrix in the table represent the starting areas of the fixation points, while columns represent the target areas.

As can be seen from Table 2, before the appearance of distance signs, the probability of the repeated fixation of familiar drivers on the area of the front window was generally less than that of unfamiliar drivers. The number of transition paths was defined as the number of paths with nonzero transition probabilities. Both familiar and unfamiliar drivers had 33 fixation transition paths.

After the presence of distance signs, the probability of the repeated staring of drivers on an area in the tunnel was greater than that before their presence, and the probability of the repeated staring of unfamiliar drivers on an area in the front window was generally greater than that of familiar drivers. When the distance sign appeared on the left or right wall of the tunnel, the probability of the familiar driver’s gaze shifting to the corresponding area increased significantly, and there were 29 or 30 gaze shift paths, respectively. The presence of distance markers had little effect on the gaze shift of unfamiliar drivers, and there were 24 (distance sign on left wall) and 31 (distance sign on right wall), respectively.

After the disappearance of the distance sign, the probability of a driver repeatedly fixating on the area of the front window was not different from before its disappearance, and was slightly less for a familiar driver than for an unfamiliar driver. When a left wall distance sign disappeared, the driver paid less attention to area 2 and more to area 5. When a right wall distance sign disappeared, the driver’s gaze shift changed little. In the stage of the disappearance of the distance marker, the gaze shift path of a familiar driver was still greater than that of an unfamiliar driver.

#### 4.2.2. Two-Step Gaze Transition Probability Matrix

If a gaze point returns to the original area after the two-step transition, it is a case of what is called the “look-back phenomenon”. The main reason for this phenomenon here is that in the current driving state, this area needs repeated attention [49]. The results of the construction of a two-step gaze transition probability matrix in three sections of a tunnel are shown in Table 3.

The process of the initial position of a fixation point returning to its original area after two steps of transfer is called backtracking. It can be seen from Table 1 that before the distance sign appeared, the driver’s two-step return transfer probability in this area was the maximum and was higher than that in other areas. Moreover, the two-step transfer probability of a familiar driver was lower than that of an unfamiliar driver. Neither type of driver had zero gaze diversion paths.

After the left wall distance sign appeared, the probability of the two-step transfer of a driver to area 2 increased significantly and was more significant for an unfamiliar driver than for a familiar driver. The driver’s probability of a return two-step transition in this area was higher than in other areas, but slightly less than before the sign appeared. The two-step shift probability of familiar drivers in this area was generally lower than that of unfamiliar drivers. The numbers of gaze diversion paths for familiar and unfamiliar drivers were 35 and 34, respectively. When the current viewpoint was in area 2—i.e., the left distance marker area—the return probability of a familiar driver was 0.8697, and that of an unfamiliar driver was 0.8816.

After the right wall distance sign appeared, the two-step transfer probability of a driver to area 5 increased significantly, and was more significant for familiar drivers than for unfamiliar drivers. The probability of the two-step transfer of a driver’s return probability in this area was greater than in other areas and was not different from that before the sign appeared. The probability of the two-step shift of familiar drivers in this area was generally less than that of unfamiliar drivers. The number of gaze transfer paths for familiar drivers did not appear to be zero, while it was 32 for unfamiliar drivers. When the current viewpoint was in area 5—i.e., the right distance marker area—the return probability of a familiar driver was 0.8673, while that of an unfamiliar driver was 0.9427.

After the disappearance of a distance sign, the driver’s return two-step transition probability in this area was still higher than that in other areas, and it was greater for unfamiliar drivers than for familiar drivers. In the stage of the disappearance of the distance marker, the gaze shift path of a familiar driver was still greater than that of an unfamiliar driver. When the left wall distance sign disappeared, the level of attention the driver gave to area 2 decreased, while that given to areas 3 and 5 increased. When the current viewpoint was in area 2—i.e., the left distance marker area—the return probability of a familiar driver in this area was 0.7034, while that of an unfamiliar driver was 0.7500. When the right wall distance sign disappeared, the driver paid more attention to areas 3 and 5. When the current viewpoint was in area 5—i.e., the right distance marker area—the return probability of a familiar driver was 0.8465, while that of an unfamiliar driver was 0.9698.

#### 4.2.3. Stationary Distribution of Markov Chains

It can be seen from Table 4 that before the appearance of distance signs, familiar drivers paid the most attention to area 5, while other areas were relatively balanced. Unfamiliar drivers also paid the most attention to area 5, followed by area 3, and least attention was paid to area 1 and area 6, which include the areas of the left rearview mirror and the dashboard. After the appearance of a left wall distance sign, the amount of attention familiar drivers gave to area 2 increased significantly, by more than 300% for unfamiliar drivers. After a right wall distance sign appeared, the stationary distribution probability of familiar drivers fixating on area 5 increased, while for unfamiliar drivers it reached 65.03%. Unfamiliar drivers paid less than 10% of their attention to the dashboard. After a distance sign disappeared, the familiar driver’s gaze shifted from the distance sign area to areas 3 and 5, respectively, while an unfamiliar driver paid more attention to these two areas than a familiar driver would.

## 5. Discussion

This study aimed to analyze the impact of exit advance guide sign settings on drivers’ eye tracking in tunnels. One- and two-step gaze transition probabilities reflect different information. In Table 2, before the distance sign appeared, the repeated gaze probability shows that it was more difficult for unfamiliar drivers to obtain information in the tunnel, and the probability of them repeatedly gazing on the target increased. After the distance sign appeared, the repeated gaze probability indicated that the appearance of a distance sign presents a greater visual recognition challenge and has greater influence on unfamiliar drivers. The fixation transfer path also decreased, and more so for unfamiliar drivers, indicating that the range of gaze was smaller and more solidified. It is consistent with the results of other studies, demonstrating that the drivers’ visual search is more concentrated [49]. When a distance sign appeared on the left tunnel wall, the amount of attention a driver gave to area 2 obviously increased and the probability of an unfamiliar driver transferring their gaze from the left rearview mirror to the distance sign area was 100%, which was not conducive to driving safety. When a distance sign appeared on the right wall, it also had a significant influence on a driver’s gaze transfer. After a distance sign disappeared, the driver had a greater probability of repeatedly gazing on the same area of the front window, and the amount of attention they gave to areas 3 and 5 increased, indicating that a driver repeatedly confirmed and searched for the exit and detected the position of a lane change to prepare for it after exiting the tunnel.

The two-step gaze transition probability can reflect continuous gaze transition behavior. In Table 1, before the appearance of the distance sign, a driver’s two-step transition probability of looking back at an area was the maximum, which is related to the unique single and closed environment of a tunnel. The two-step probability of a familiar driver transitioning their gaze was lower than that of unfamiliar drivers, which indicates that unfamiliar drivers must repeatedly confirm this field. After the appearance of a left wall distance sign, the two-step probability of a driver transitioning their gaze to area 2 increased. Due to the unfamiliar driving environment, unfamiliar drivers were more significant than the familiar drivers. When the current viewpoint was in area 2—i.e., the left distance sign area—the look-back probability of familiar drivers was 0.8697, while that of unfamiliar drivers was 0.8816. This is because unfamiliar drivers make greater efforts to recognize and read distance signs. It is consistent with the results of other studies [15,55]. The same occurred after a right wall distance sign appeared. The driver’s two-step transition probability of looking back in this area was higher than in other areas, but slightly lower than that before the sign appeared. Unfamiliar drivers need to repeatedly confirm the sign area. Familiar drivers have more visual transfer paths than unfamiliar drivers, indicating that, in addition to the sign, other areas also had a significant impact on the familiar drivers. After a distance sign disappeared, the driver’s two-step probability of transitioning their gaze was still high. To adapt to the white-hole effect and find the exit, although both groups of drivers paid more attention to areas 3 and 5, the unfamiliar drivers were more significant.

In Table 3, drivers paid the most attention to area 5 because they were looking for the exit, which are on the right side of the highway in China. It is consistent with the results of other studies [56]. Familiar drivers had a more balanced fixation distribution in each area. Unfamiliar drivers paid less attention to the dashboard. When a driver paid more attention to unfamiliar signs, the corresponding increase in attention given to the dashboard was 10%. This shows that the setting of traffic signs is more effective for familiar drivers and can attract attention without affecting driving safety. After a right wall distance sign appeared, the results show that the appearance of the sign had a significant impact on the driver. As unfamiliar drivers were not familiar with the experimental environment, they paid too much attention to the sign, which indicates that they made greater effort to read and recognize the sign information and ignored other areas. Babić [8] found that drivers used more time to realize unfamiliar signs. Yang [57] showed that drivers who were unfamiliar had a cognitive load based on eye movement. After the sign indicating distance disappeared, the driver’s gaze shifted from the distance sign to areas 3 and 5, in that order, and unfamiliar drivers paid more attention to these two areas than familiar drivers. This is because a driver who identifies the location and distance information signs knows that the ramp is on the right side of the tunnel, is aware of the white-hole effect of coming out of the tunnel, and pays more attention to the right wall and vault area of the tunnel and searches for the exit ramp. Familiar drivers paid more attention than unfamiliar drivers to vehicle speed to ensure driving safety.

At any stage, the probability of the repeated gaze and two-step back-shift of unfamiliar drivers was generally higher than that of familiar drivers because it is more difficult for unfamiliar drivers to obtain information. The number of transfer paths of unfamiliar drivers was lower than that of familiar drivers, indicating that the latter had wider vision. Similar results have been found in other studies [58]. When signs appeared, familiar drivers paid attention to them and took into account a safe driving speed, while unfamiliar drivers were too concerned about the sign. When signs disappeared, the gazes of all drivers transferred from area 3 to area 5, but the attention unfamiliar drivers gave to these areas was higher than that of familiar drivers, and they ignored vehicle speed, indicating lower safety awareness and driving behavior. It is consistent with the results of other studies [56].

The drivers recruited in the experiments are not representative of all drivers. In addition, different lane conditions and types may affect a driver’s gaze transition, so future research requires a wider range of experimental data.

## 6. Conclusions

We proposed that separate guide signs in tunnel exits be placed by using an eye tracker and indoor simulation test platform. By changing the placement positions of signs for familiar and unfamiliar drivers, we obtained the setting effects for separate guide signs with different drivers. The results showed that separate guide signs had significant effects on the eye fixation area. At any stage, the probability of unfamiliar drivers repeatedly gazing at an area and performing two-step back-shift exceeded that of familiar drivers, showing that it is more difficult for unfamiliar drivers to obtain information. This is consistent with the conclusions of other research. The number of transfer paths of unfamiliar drivers was lower than that of familiar drivers, which indicates that the latter had wider vision and therefore more conducive to driving safety. When signs appeared, familiar drivers paid attention to them and took into account a safe driving speed. Unfamiliar drivers were too concerned about the sign. When signs disappeared, the gazes of familiar and unfamiliar drivers were transferred from area 3 to area 5, but the attention unfamiliar drivers paid to these two areas was higher than that of familiar drivers, and they ignored vehicle speed, indicating that the safety awareness and driving behavior of unfamiliar drivers are lower than those of familiar drivers. Some scholars have studied how to set up exit advance guide signs in highway tunnels. No research has been carried out on evaluating them according to the eye-tracking of familiar and unfamiliar drivers. Therefore, this research work could provide a reasonable reference for exit advance guide sign design in highway tunnels. The main limitation of the present study lies in the fact that it was limited to the use of the same lighting; however, different tunnel lighting factors could be considered in future work.

## Figures and Tables

**Table 1:** One-step gaze transition probability matrix in tunnel.

Sections		Familiar Drivers	Unfamiliar Drivers
Before sign appears		$\left[\begin{array}{l} 0.9180\;0.0257\;0.0000\;0.0050\;0.0051\;0.0462\\ 0.0090\;0.8541\;0.0565\;0.0689\;0.0034\;0.0081\\ 0.0017\;0.0951\;0.7963\;0.0129\;0.0884\;0.0053\\ 0.0000\;0.1780\;0.0298\;0.5624\;0.1176\;0.1121\\ 0.0000\;0.0034\;0.0445\;0.0382\;0.9137\;0.0006\\ 0.0243\;0.0211\;0.0035\;0.1470\;0.0175\;0.7867 \end{array}\right]$	$\left[\begin{array}{l} 0.9106\;0.0317\;0.0189\;0.0000\;0.0199\;0.0189\\ 0.0108\;0.8433\;0.1175\;0.0223\;0.0000\;0.0061\\ 0.0000\;0.0537\;0.8908\;0.0035\;0.0521\;0.0000\\ 0.0344\;0.0510\;0.0122\;0.6441\;0.0854\;0.1729\\ 0.0000\;0.0028\;0.0602\;0.0143\;0.9205\;0.0021\\ 0.0170\;0.0346\;0.0000\;0.2587\;0.0345\;0.6552 \end{array}\right]$
After sign appears	sign on left wall	$\left[\begin{array}{l} 0.7827\;0.1957\;0.0000\;0.0000\;0.0000\;0.0216\\ 0.0068\;0.8332\;0.0765\;0.0467\;0.0139\;0.0229\\ 0.0000\;0.0883\;0.6917\;0.0825\;0.1340\;0.0034\\ 0.0000\;0.1540\;0.0879\;0.5979\;0.1218\;0.0384\\ 0.0000\;0.0141\;0.0961\;0.0309\;0.8569\;0.0020\\ 0.0077\;0.0055\;0.0000\;0.0242\;0.0075\;0.9551 \end{array}\right]$	$\left[\begin{array}{l} 0.0000\;1.0000\;0.0000\;0.0000\;0.0000\;0.0000\\ 0.0015\;0.9039\;0.0354\;0.0201\;0.0370\;0.0021\\ 0.0000\;0.0648\;0.8060\;0.0105\;0.0087\;0.1096\\ 0.0000\;0.1748\;0.0550\;0.5963\;0.0366\;0.1371\\ 0.0000\;0.0247\;0.0699\;0.0047\;0.9007\;0.0000\\ 0.0000\;0.0153\;0.0000\;0.0795\;0.0000\;0.9053 \end{array}\right]$
	sign on right wall	$\left[\begin{array}{l} 0.8789\;0.0494\;0.0000\;0.0717\;0.0000\;0.0000\\ 0.0388\;0.6246\;0.0677\;0.1058\;0.1054\;0.0577\\ 0.0055\;0.0072\;0.8346\;0.0102\;0.1374\;0.0051\\ 0.0000\;0.2345\;0.0512\;0.1633\;0.4694\;0.0816\\ 0.0000\;0.0100\;0.0423\;0.0194\;0.9259\;0.0023\\ 0.0000\;0.0422\;0.0041\;0.0024\;0.0124\;0.9388 \end{array}\right]$	$\left[\begin{array}{l} 0.9272\;0.0346\;0.0000\;0.0064\;0.0000\;0.0318\\ 0.0203\;0.9536\;0.0158\;0.0016\;0.0029\;0.0058\\ 0.0000\;0.0075\;0.8951\;0.0140\;0.0804\;0.0030\\ 0.0000\;0.1348\;0.0261\;0.5870\;0.2174\;0.0348\\ 0.0005\;0.0005\;0.0128\;0.0108\;0.9728\;0.0026\\ 0.0489\;0.0072\;0.0000\;0.0550\;0.0449\;0.8440 \end{array}\right]$
After sign disappears	sign on left wall	$\left[\begin{array}{l} 0.8576\;0.1034\;0.0000\;0.0145\;0.0000\;0.0245\\ 0.0133\;0.8355\;0.1240\;0.0197\;0.0009\;0.0066\\ 0.0018\;0.0625\;0.9012\;0.0015\;0.0329\;0.0000\\ 0.0535\;0.1739\;0.0770\;0.2174\;0.3913\;0.0870\\ 0.0000\;0.0154\;0.0309\;0.0112\;0.9425\;0.0000\\ 0.0330\;0.0230\;0.0000\;0.0115\;0.0000\;0.9325 \end{array}\right]$	$\left[\begin{array}{l} 0.9543\;0.0000\;0.0457\;0.0000\;0.0000\;0.0000\\ 0.0235\;0.8447\;0.1215\;0.0035\;0.0069\;0.0000\\ 0.0000\;0.0647\;0.8570\;0.0024\;0.0759\;0.0000\\ 0.0000\;0.0000\;0.5300\;0.0000\;0.3233\;0.1567\\ 0.0000\;0.0078\;0.1056\;0.0047\;0.8820\;0.0000\\ 0.0000\;0.0000\;0.0000\;0.0000\;0.0427\;0.9573 \end{array}\right]$
	sign on right wall	$\left[\begin{array}{l} 0.9306\;0.0317\;0.0159\;0.0000\;0.0059\;0.0159\\ 0.0158\;0.8303\;0.1055\;0.0323\;0.0000\;0.0161\\ 0.0000\;0.0487\;0.8968\;0.0010\;0.0536\;0.0000\\ 0.0224\;0.0630\;0.0122\;0.6341\;0.0854\;0.1829\\ 0.0000\;0.0121\;0.0505\;0.0146\;0.9207\;0.0021\\ 0.0172\;0.0349\;0.0000\;0.2582\;0.0344\;0.6552 \end{array}\right]$	$\left[\begin{array}{l} 0.7143\;0.1805\;0.0000\;0.0000\;0.0576\;0.0476\\ 0.0273\;0.9306\;0.0158\;0.0263\;0.0000\;0.0000\\ 0.0000\;0.0083\;0.9707\;0.0126\;0.0084\;0.0000\\ 0.0052\;0.0162\;0.0175\;0.8734\;0.0292\;0.0584\\ 0.0000\;0.0000\;0.0069\;0.0106\;0.9814\;0.0012\\ 0.0000\;0.0000\;0.0000\;0.1468\;0.0160\;0.8372 \end{array}\right]$

**Table 2:** Classification of gaze targets in each area.

Area Number	Area of Interest	Area Description
1	Left area	Area displayed by the left rearview mirror
2	Left side	Area on the left-hand side (e.g., distance sign, left wall of the tunnel)
3	Top	Area towards the top of the tunnel (e.g., location sign)
4	Road	Area of the road in the tunnel
5	Right area	Area towards the right-hand side (e.g., distance sign, right mirror, left wall of the tunnel)
6	Dashboard	Dashboard

**Table 3:** Two-step gaze transition probability matrix in a tunnel.

Section		Familiar Drivers	Unfamiliar Drivers
Before sign appears		$\left[\begin{array}{l} 0.8545\;0.0471\;0.0020\;0.0162\;0.0107\;0.0695\\ 0.0174\;0.7462\;0.0951\;0.0994\;0.0194\;0.0225\\ 0.0085\;0.1574\;0.6382\;0.0304\;0.1538\;0.0117\\ 0.0069\;0.2552\;0.0557\;0.3503\;0.1785\;0.1534\\ 0.0012\;0.0194\;0.0763\;0.0512\;0.8418\;0.0101\\ 0.0659\;0.0514\;0.0118\;0.2005\;0.0346\;0.6358 \end{array}\right]$	$\left[\begin{array}{l} 0.8268\;0.1112\;0.0168\;0.0204\;0.0028\;0.0220\\ 0.0358\;0.7641\;0.1150\;0.0508\;0.0302\;0.0041\\ 0.0016\;0.0652\;0.8455\;0.0071\;0.0784\;0.0022\\ 0.0074\;0.1198\;0.0309\;0.5354\;0.2203\;0.0862\\ 0.0001\;0.0065\;0.0412\;0.0311\;0.9157\;0.0054\\ 0.0439\;0.0315\;0.0223\;0.2381\;0.0950\;0.5692 \end{array}\right]$
After sign appears	sign on left wall	$\left[\begin{array}{l} 0.6100\;0.3358\;0.0073\;0.0066\;0.0018\;0.0385\\ 0.0165\;0.8697\;0.0451\;0.0352\;0.0248\;0.0087\\ 0.0007\;0.1564\;0.4981\;0.1164\;0.2196\;0.0087\\ 0.0039\;0.2450\;0.1283\;0.3736\;0.2001\;0.0491\\ 0.0000\;0.0245\;0.1526\;0.0663\;0.7514\;0.0052\\ 0.0078\;0.0180\;0.0038\;0.0412\;0.0160\;0.9131 \end{array}\right]$	$\left[\begin{array}{l} 0.0039\;0.9438\;0.0352\;0.0136\;0.0027\;0.0008\\ 0.0024\;0.8816\;0.0651\;0.0304\;0.0082\;0.0132\\ 0.0012\;0.1869\;0.7222\;0.0176\;0.0671\;0.0050\\ 0.0008\;0.2753\;0.0871\;0.3724\;0.0575\;0.2069\\ 0.0000\;0.0366\;0.1206\;0.0240\;0.8167\;0.0022\\ 0.0000\;0.0233\;0.0061\;0.1359\;0.0032\;0.8315 \end{array}\right]$
	sign on right wall	$\left[\begin{array}{l} 0.7714\;0.0822\;0.0168\;0.0749\;0.0465\;0.0082\\ 0.0462\;0.4303\;0.0954\;0.0897\;0.2386\;0.0998\\ 0.0092\;0.0155\;0.7034\;0.0131\;0.2472\;0.0116\\ 0.0078\;0.1861\;0.0943\;0.0592\;0.5485\;0.1042\\ 0.0026\;0.0292\;0.0760\;0.0175\;0.8673\;0.0074\\ 0.0012\;0.0553\;0.0105\;0.0173\;0.0324\;0.8833 \end{array}\right]$	$\left[\begin{array}{l} 0.8236\;0.0829\;0.0124\;0.0125\;0.0020\;0.0675\\ 0.0389\;0.9102\;0.0110\;0.0296\;0.0088\;0.0015\\ 0.0005\;0.0135\;0.8026\;0.0222\;0.1554\;0.0058\\ 0.0028\;0.0578\;0.0441\;0.4766\;0.3636\;0.0551\\ 0.0022\;0.0034\;0.0210\;0.0265\;0.9427\;0.0051\\ 0.0814\;0.0203\;0.0021\;0.0852\;0.0941\;0.7169 \end{array}\right]$
After sign disappears	sign on left wall	$\left[\begin{array}{l} 0.6671\;0.1828\;0.0189\;0.0387\;0.0268\;0.0657\\ 0.0075\;0.7034\;0.2199\;0.0221\;0.0293\;0.0196\\ 0.0054\;0.1082\;0.8217\;0.0021\;0.0620\;0.0006\\ 0.0467\;0.1972\;0.1350\;0.0591\;0.4586\;0.1034\\ 0.0015\;0.0414\;0.0612\;0.0186\;0.8763\;0.0010\\ 0.0429\;0.0483\;0.0022\;0.0137\;0.0026\;0.8903 \end{array}\right]$	$\left[\begin{array}{l} 0.9104\;0.0011\;0.0658\;0.0091\;0.0136\;0.0000\\ 0.0073\;0.7500\;0.2194\;0.0075\;0.0146\;0.0012\\ 0.0009\;0.0775\;0.7672\;0.0017\;0.1523\;0.0004\\ 0.0000\;0.0356\;0.4680\;0.0038\;0.3499\;0.1427\\ 0.0000\;0.0280\;0.1782\;0.0046\;0.7808\;0.0084\\ 0.0000\;0.0014\;0.0032\;0.0003\;0.0787\;0.9164 \end{array}\right]$
	sign on right wall	$\left[\begin{array}{l} 0.8585\;0.0676\;0.0230\;0.0054\;0.0303\;0.0152\\ 0.0293\;0.7060\;0.1728\;0.0511\;0.0099\;0.0309\\ 0.0026\;0.0760\;0.8148\;0.0085\;0.0968\;0.0013\\ 0.0467\;0.0923\;0.0328\;0.4507\;0.1301\;0.2474\\ 0.0013\;0.0064\;0.1232\;0.0200\;0.8465\;0.0026\\ 0.0358\;0.0665\;0.0093\;0.3342\;0.0775\;0.4767 \end{array}\right]$	$\left[\begin{array}{l} 0.5055\;0.3134\;0.0131\;0.0135\;0.0807\;0.0738\\ 0.0454\;0.8744\;0.0304\;0.0466\;0.0020\;0.0012\\ 0.0012\;0.0127\;0.9411\;0.0268\;0.0173\;0.0009\\ 0.0076\;0.0300\;0.0357\;0.7721\;0.0542\;0.1004\\ 0.0000\;0.0017\;0.0104\;0.0159\;0.9698\;0.0022\\ 0.0008\;0.0026\;0.0026\;0.2504\;0.0398\;0.7038 \end{array}\right]$

**Table 4:** Stationary distribution of the Markov chains in a tunnel.

Familiar/Unfamiliar	Before Sign Appears	After Sign Appears		After the Sign Disappears	
		Sign on Left Wall	Sign on Right Wall	Sign on Left Wall	Sign on Right Wall
Familiar drivers	$\left[\begin{array}{l} 0.0681\\ 0.2595\\ 0.1503\\ 0.1012\\ 0.3354\\ 0.0855 \end{array}\right]$	$\left[\begin{array}{l} 0.0160\\ 0.4440\\ 0.1284\\ 0.0832\\ 0.2125\\ 0.1159 \end{array}\right]$	$\left[\begin{array}{l} 0.0272\\ 0.0448\\ 0.1816\\ 0.0328\\ 0.6128\\ 0.1009 \end{array}\right]$	$\left[\begin{array}{l} 0.0165\\ 0.1893\\ 0.3772\\ 0.0151\\ 0.3456\\ 0.0562 \end{array}\right]$	$\left[\begin{array}{l} 0.0587\\ 0.1495\\ 0.3576\\ 0.0607\\ 0.3207\\ 0.0528 \end{array}\right]$
Unfamiliar drivers	$\left[\begin{array}{l} 0.0380\\ 0.1486\\ 0.2438\\ 0.0671\\ 0.4779\\ 0.0245 \end{array}\right]$	$\left[\begin{array}{l} 0.0029\\ 0.5625\\ 0.2012\\ 0.0440\\ 0.1251\\ 0.0643 \end{array}\right]$	$\left[\begin{array}{l} 0.0581\\ 0.1067\\ 0.0975\\ 0.0397\\ 0.6504\\ 0.0476 \end{array}\right]$	$\left[\begin{array}{l} 0.0357\\ 0.1490\\ 0.4527\\ 0.0053\\ 0.3441\\ 0.0132 \end{array}\right]$	$\left[\begin{array}{l} 0.0290\\ 0.0804\\ 0.2336\\ 0.1620\\ 0.4392\\ 0.0558 \end{array}\right]$

## References

[1] Wga X., Pb L., Sb D., Ha K., Sb N. (2006). Recognition of traffic signs based on their colour and shape features extracted using human vision models. J. Vis. Commun. Image Represent..

[2] Lambert L.D., Fleury M. (1994). Age, cognitive style, and traffic signs. Perceptual Motor Skills.

[3] Liu B.H., Sun L.H., Rong J. (2011). Driver’s visual cognition behaviors of traffic signs based on eye movement parameters. J. Transp. Syst. Eng. Inf. Technol..

[4] Du Z.G., Tang Y.H., Xie J., Li P.F. New research on improved highway tunnel based on visual illusion. Proceedings of the Transportation Reform and Change-Equity, Inclusiveness, Sharing, and innovation-Proceeding of the 17th COTA Interation Conference of Transportation Professionals.

[5] Bassan S. (2016). Overview of traffic safety aspects and design in road tunnels. Iatss Res..

[6] (2014). Investigation Report of Particularly Hazardous Road Traffic Accident of Yanhou Tunnel of Jincheng of Shanxi Province.

[7] Japan Today. 2 dead, 70 injured after 12-vehicle accident in Hiroshima tunnel. Japan Today: Japan News and Discussion.

[8] Babić D., Dijanić H., Jakob L., Babić D., Garcia-Garzon E. (2020). Driver eye movements in relation to unfamiliar traffic signs: An eye tracking study. Appl. Ergon..

[9] David S., Robert E., Dewar H.S., Lidia Z. (2003). Traffic sign symbol comprehension: A cross-cultural study. Ergonomics.

[10] Treisman A. (1986). Features and objects in visual processing. Sci. Am..

[11] Babic D., Babić D., Ščukanec A. The impact of road familiarity on the perception of traffic signs—Eye tracking case study. Proceedings of the International Conference on Environmental Engineering.

[12] Ben-Bassat T., Shinar D. (2006). Ergonomic guidelines for traffic sign design increase sign comprehension. Hum. Factors.

[13] United Nations World Tourism Organization UNWTO Annual Report 2015. http://www2.unwto.org/publication/unwto-annual-report-2015.

[14] Aty M.A., Radwan A.E. (1998). Demographic Factors and Traffic Crashes: Part i-Descriptive Statistics and Models.

[15] Stephanie H., Sonia C. An Eye-tracking valuation of driver distraction and unfamiliar road signs. Proceedings of the 8th International Conference on Automotive User Interfaces and Interactive Vehicular Applications.

[16] Shang T., Lu H., Wu P., Lu X. (2021). Method of setting exit advance guide signs in highway tunnels based on the driver’s eye movement with markov chains. IEEE Access.

[17] Tatler B.W., Kirtley C., Macdonald R.G., Mitchell K., Savage S.W. (2014). The Active Eye: Perspectives on Eye Movement Research. Current Trends in Eye Tracking Research.

[18] Board T.R. (1998). Managing Speed: Review of Current Practices for Setting and Enforcing Speed Limits.

[19] National Academies of Science (2000). Highway Capacity Manual.

[20] Garber N.J., Gadiraju R. (1989). Factors affecting speed variance and its influence on accidents. Transp. Res. Rec. J. Transp. Res. Board.

[21] Shrira I., Noguchi K. (2016). Traffic fatalities of drivers who visit urban and rural areas: An exploratory study. Transp. Res. Part F Traffic Psychol. Behav..

[22] Du Z.G., Pan X.D. Application research of visual cognition probabilistic model on urban tunnel’s sign. Proceedings of the 2009 International Conference on Measuring Technology and Mechatronics Automation.

[23] Hong I., Iwasaki M., Furuichi T., Kadoma T. Eye movement and driving behavior in curved section passages of urban motorway. Proceedings of the TRB 2005 Annual Meeting CD-ROM.

[24] Mele M.L., Federici S. (2012). Gaze and eye-tracking solutions for psychological research. Cogn. Process..

[25] Martens M.H., Fox M. (2007). Do familiarity and expectations change perception? Drivers’ glances and response to changes. Transp. Res. Part F Traffic Psychol. Behav..

[26] Donmez B., Boyle L.N., Lee J.D. (2009). Designing Feedback to Mitigate Distraction.

[27] Maltz M., Shinar D. (2007). Imperfect in-vehicle collision avoidance warning systems can aid distracted drivers. Hum. Factors.

[28] Thompson C., Sabik M. (2018). Allocation of attention in familiar and unfamiliar traffic scenarios. Transp. Res. Part F Traffic Psychol. Behav..

[29] Ben-Bassat T. (2019). Are ergonomically designed road signs more easily learned?. Appl. Ergon..

[30] Intini P., Colonna P., Berloco N., Ranieri V. The impact of route familiarity on drivers’ speeds, trajectories and risk perception. Proceedings of the 17th International Conference Road Safety on Five Continents (RS5C 2016).

[31] Yanko M.R., Spalek T.M. (2013). Route familiarity breeds inattention: A driving simulator study. Accid. Anal. Prev..

[32] Hurtado S., Chiasson S. An eye-Tracking Evaluation of Driver Distraction and Road Signs. Proceedings of the 8th International Conference on Automotive User Interfaces and Interactive Vehicular Applications.

[33] Zwahlen H., Russ A., Roth J., Schnell T. (2003). Effectiveness of ground-mounted diagrammatic advance guide signs for freeway entrance ramps. Transp. Res. Rec. J. Transp. Res. Board.

[34] Song Y. Study on quantitative evaluation of range of advance entrance direction sign of expressway based on auto navigation data analysis. Proceedings of the Cota International Conference of Transportation Professionals.

[35] Huang L., Zhao X., Li Y., Ma J., Wang Y. (2020). Optimal design alternatives of advance guide signs of closely spaced exit ramps on urban expressways. Accid. Anal. Prev..

[36] Fang L., Min H., Teng Z., Feng M. (2017). A guide sign optimization problem for an added road based on bird mating optimizer. International Conference on Swarm Intelligence.

[37] Han B., Yu L., Tong Z., Xie D., Liang Z. (2018). Locating speed limit signs for freeway tunnel entrance and exit. International Conference on Swarm Intelligence.

[38] Pan X.D., Guo X.B., Du Z.G. The attentive point of driver’s watch and the research of traffic safety experiment. Proceedings of the 7th Annual Conference Academic Collections of the Shanghai Road Academy.

[39] (2018). Design Specification for Highway Alignment, JTG D20-2017.

[40] (2019). Specifications for Design of Highway Tunnel, JTG 3370.1-2018.

[41] (2009). Road Traffic Signs and Markings, GB5768.2-2009.

[42] (2014). Guidelines for Design of Highway Grade-Separated Intersections, JTG/T D21-2014.

[43] Wang W., Liu B. (2011). Automatic Recognition Algorithm of Traffic Signs in Road Tunnel.

[44] Upchurch J., Fisher D., Carpenter R., Dutta A. (2002). Freeway guide sign design with driving simulator for central artery-tunnel: Boston, massachusetts. Transp. Res. Rec. J. Transp. Res. Board.

[45] Yan B., Zhou J.B., Wang L. (2013). Effectiveness of traffic sign setting in adjacent tunnel exit. Proc.-Soc. Behav. Sci..

[46] Song C.C., Guo Z.Y., Qiao Y.D. Location of signs at tunnel entrance and layout combination of signs and markings. Proceedings of the 17th COTA conference International Conference of Transportation Professionals (CICTP2017).

[47] Liechty J., Pieters R., Wedel M. (2003). Global and local covert visual attention: Evidence from a bayesian hidden markov model. Psychometrika.

[48] Krejtz K., Duchowski A., Szmidt T., Krejtz I., Villalobos N. (2015). Gaze transition entropy. ACM Trans. Appl. Percept..

[49] Jspa B., Cww B., Rf C., Wy C. (2020). Extraction of parameters for lane change intention based on driver’s gaze. Saf. Sci..

[50] Li Y., Wang F., Ke H., Wang L.L., Xu C.C. (2019). A Driver’s Physiology Sensor-Based Driving Risk Prediction Method for Lane-Changing Process Using Hidden Markov Model. Sensors.

[51] Zhou Z., Ma J., Lu T., Li G., Tan T. (2020). An evaluation method for visual search stability in urban tunnel entrance and exit sections based on markov chain. IEEE Access.

[52] Pan S., Guo T., Shao Y.F. (2018). Study on drivers’ fixation transfer characteristics in urban tunnel environment. Chin. J. Saf. Sci..

[53] Wen J.Z., Du Z.G., Wang S.S. (2019). Studies on the distribution and transfer of fixation points in optical long tunnels with small radius in mountainous areas. Traffic Inf. Saf..

[54] Schieber F., Goodspeed C.H. Nighttime conspicuity of highway signs as a function of sign brightness, background complexity and age of Observer. Proceedings of the Human Factors and Ergonomics Society Annual Meeting.

[55] Michael P.P., Srinivas R.G., Bahar D., Lingtao W., Mohammadali S. (2019). Familiar versus unfamiliar drivers on curves: Naturalistic data study. Transp. Res. Record.

[56] Tan H. (2016). Safety Analysis on the Interchange Exit Ramp of Mountainous Urban Road. Master’s Thesis.

[57] Yang Y.Q., Chen J.Y., Easa S.M., He Z.Y., Yin D.N., Zheng X.Y. (2021). Internal causes of return trip effect based on eye movement and EEG indices. Transp. Res. Part F Traffic Psychol. Behav..

[58] Pan S., Guo T.Y., Shao F., Xu Q. (2018). Research on fixation transfer characteristics of drivers driving through urban tunnel. China Saf. Sci. J..

